# Laboratory information- management the CALS approach

**DOI:** 10.1155/S1463924685000414

**Published:** 1985

**Authors:** John Boother

**Affiliations:** Beckman Instruments Progress Road Sands Industrial Estate Buckinghamshire High Wycombe HP12 4JL UK


Journal of Automatic Chemistry/*Journal of Clinical Laboratory Automation*, Vol. 7, No. 4 (October–December 1985), pp. 185–191

### Laboratory information management – the CALS approach

**John Boother**
*Beckman Instruments, Progress Road, Sands Industrial Estate, High Wycombe, Buckinghamshire HP12 4JL, UK*

Because laboratories typically process large numbers of samples every day, information management is a critical task. With a growing work-load in the laboratory, the chances for errors of omission and data transcription increase, as does the amount of time devoted to paperwork. Moreover periodic review and analysis of the progress and quality of laboratory work becomes increasingly important. In fact, a laboratory can be only as good as its ability to manage its final product – the information it produces. Laboratory productivity, accuracy and control are usually greatly increased with improved information processing capabilities.

Use of the CALS Lab Manager System helps to organize and standardize routine operations within the laboratory. Consistent, non-redundant entry of product specifications, data acquisition, analysis methods and test specification limits encourages accuracy and repeatability. Automatic method revision control with security, time and date and user ID stamping encourages timely updating with minimum paperwork and loss of control. Rapid, inexpensive access to all information minimizes guesswork and encourages informed accuracy.

### Overview

The CALS Lab Manager System is a laboratory information management tool, which facilitates the efficient and accurate flow of information through the laboratory environment. As an integrated information management system, Lab Manager monitors the status of all samples being processed by the laboratory, records analysis results either manually or from on-line instrumentation, generates a variety of reports including multi-coloured plots and enforces adherence to local laboratory practice and procedures.

The system completely tracks all samples through every step of laboratory processing; it also maintains a complete directory of the specifications or test protocols for all products handled by the laboratory, as well as a directory of all defined tests. All data recorded for each sample is stored in the Lab Manager data-base on-line without time lags. The flexible inquiry facility allows both selective retrieval and customized reporting of all stored data. The archiving facility provides a simple and rapid means of saving and recalling raw data, results and methods on low cost media.

To meet the specific and evolving needs of an individual laboratory, the Lab Manager program incorporates many configurable and optional features. Within limits, each standard system may be configured and customized without additional software and therefore without additional cost.

### *Features and facilities*

Complete laboratory information automation.
User-friendly operation including configurable, personalized menus and full screen data entry.
Configurable to current and future requirements.
Extensive use of user-controlled dictionaries and tables.
Configurable data-base contents.
Prospective or retrospective sample log-in.
Customized adhesive label generation.
Yes/no, free text, choice, quantitative value and on-line data acquisition test types.
Configurable, user-specified sample status reports.
Configurable, user-specified 'pending test' status reports.
User-specified calculation dictionary to automatically compute secondary values.
User-specified product specifications dictionary automatically schedules routine tests and limits for samples.
Very high level, *ad hoc* data retrieval and report formatting capabilities including sorting, headings, footings and margin control.
Complete data-base archiving and recall using low-cost media or telecommunications.
Class oriented, rather than hierarchical level, security system for function control and data integrity.
Configurable test result validation and sample result approval step.

### *User-friendly operation*

All Lab Manager operation is directed in a conversational user-friendly manner. If desired, Lab Manager functions may be invoked from simple, user-specified menus. Test results may be entered using either prompted or an optional 'full screen' mode.

The Lab Manager menu structure is particularly flexible. Each laboratory may specify its own menu structure, including the descriptive text and position of each function on a screen. Each user may optionally utilize a customized menu structure, which is organized specifically for his/her most frequently used system capabilities. This capability minimizes the number of screens required to accomplish most tasks and increases productivity. The menu structure interacts with the system security feature, so only functions available to the user's authority class are displayed. Additionally, any authorized function may be invoked by entry of a simple two-character code, regardless of the menu currently displayed. This short-

© 1985 Beckman-RIIC Ltd cut is often used by experienced operators who do not need a complete menu.

If the full screen data entry mode is selected, each screen format may be specified by the laboratory to match local vocabulary and requirements. During construction of full screen formats, fields may be arbitrarily positioned, labelled with headings, footings and other decorations and assigned any of the available visual attributes, including colour, for emphasis.

Additionally, the optional RUNSHEET program combines the functions of work-list generation and test scheduling with minimal user interaction.

The combination of these features provides for a user-friendly operational environment, which may be readily adapted to special laboratory requirements. Not only do these features assist at every step, but also minimize total keystrokes and operator interaction.

*Flexibility*

Features of Lab Manager which can be easily configured include prompting messages, displayed messages, the organization of commands into menus, screen formats, the organization of specific files known as dictionaries, dictionary contents, and the elimination or inclusion of optional tasks (see figure 1). Products may be linked with standard testing sequences, validation limits may be defined and report formats may be specified. Most importantly, the set of data items stored on the data-base for samples and tests are completely configurable and determined in accordance to local needs and vocabulary. All of these items, and many more, may be initially selected by the laboratory and subsequently changed as requirements, instrumentation and methods evolve.

*System and data security*

The CALS System itself imposes a restriction on execution of commands and programs from a given terminal. In this way remote terminals may be prohibited from doing anything other than their allowed functions and programs.

In addition, Lab Manager supports up to 16 classes of authority for users. Access to specific information may therefore be limited to personnel belonging to the appropriate security class. Powerful commands, such as those which delete information or allow file modification, can be easily restricted. The security classes are initially defined during system installation and subsequently refined by the laboratory. Usually, each laboratory's system manager assigns and maintains user IDs, passwords, and security classes necessary for log-on and process execution.

The class-oriented security mechanism is more flexible and complete than simple hierarchical or password systems. Use of classes allows real control over the functions authorized to each system user and is essential to Good Laboratory Practice (GLP) compliance and proper laboratory management.

*Productivity and cost-effectiveness*

The productivity of Lab Manager use becomes very clear after even a short period of operation:

(1) The Lab Manager System is very configurable – this allows conformance to established laboratory procedures, methods, and data elements, resulting in minimal operational retraining. This configurability allows the system to evolve and grow with the laboratory.
(2) The Lab Manager operates in a conversational, user-friendly fashion. The system prompts for all entries and validates each before proceeding. This allows errors to be corrected at the most cost-effective moment. All Lab Manager functions may be optionally organized into menus or activated by short, single-line command strings once familiarity is achieved.
(3) The Lab Manager provides a complete set of laboratory information management tools. Database contents are completely determined by laboratory requirements. Virtually any type of laboratory test result may be entered, minimizing the potential of 'partial automation' with retained paperwork and manual procedures. Similarly, the report facility is completely under laboratory control and does not require extensive programming.
(4) A flexible calculation dictionary essentially eliminates manual computation of secondary calculations, minimizing labour and errors.

### Operational task sequence

While Lab Manager directs and maintains the flow of analyses in accordance with the specific tasks of laboratories in a variety of industries, it does use a defined sequence of operations as its basic structure for managing information. This sequence is represented in figure 1.

*Sample log-in*

The sample log-in step involves registering the sample with the system by assigning it a unique identification number (ID). Lab Manager will ask the user for this ID, using prompts worded according to the type of work done in the lab. The structure of the ID is completely configurable, and may be hierarchical. The ID may be assigned by the user, sequentially assigned by the system, or even suggested by Lab Manager prompting.

During log-in, an adhesive label containing any or all log-in data may be printed. Optionally, multiple copies may be printed to label split sample containers.

*Testing*

The testing step represents most of the actual work done in laboratories. Lab Manager is designed to accept manual input from laboratory personnel, as they perform various tests, by using a series of appropriate prompts to request data. These tests can be manual operations, ranging from simply recording freely worded comments, or sample descriptions, to recording quantitative results from complex assays.

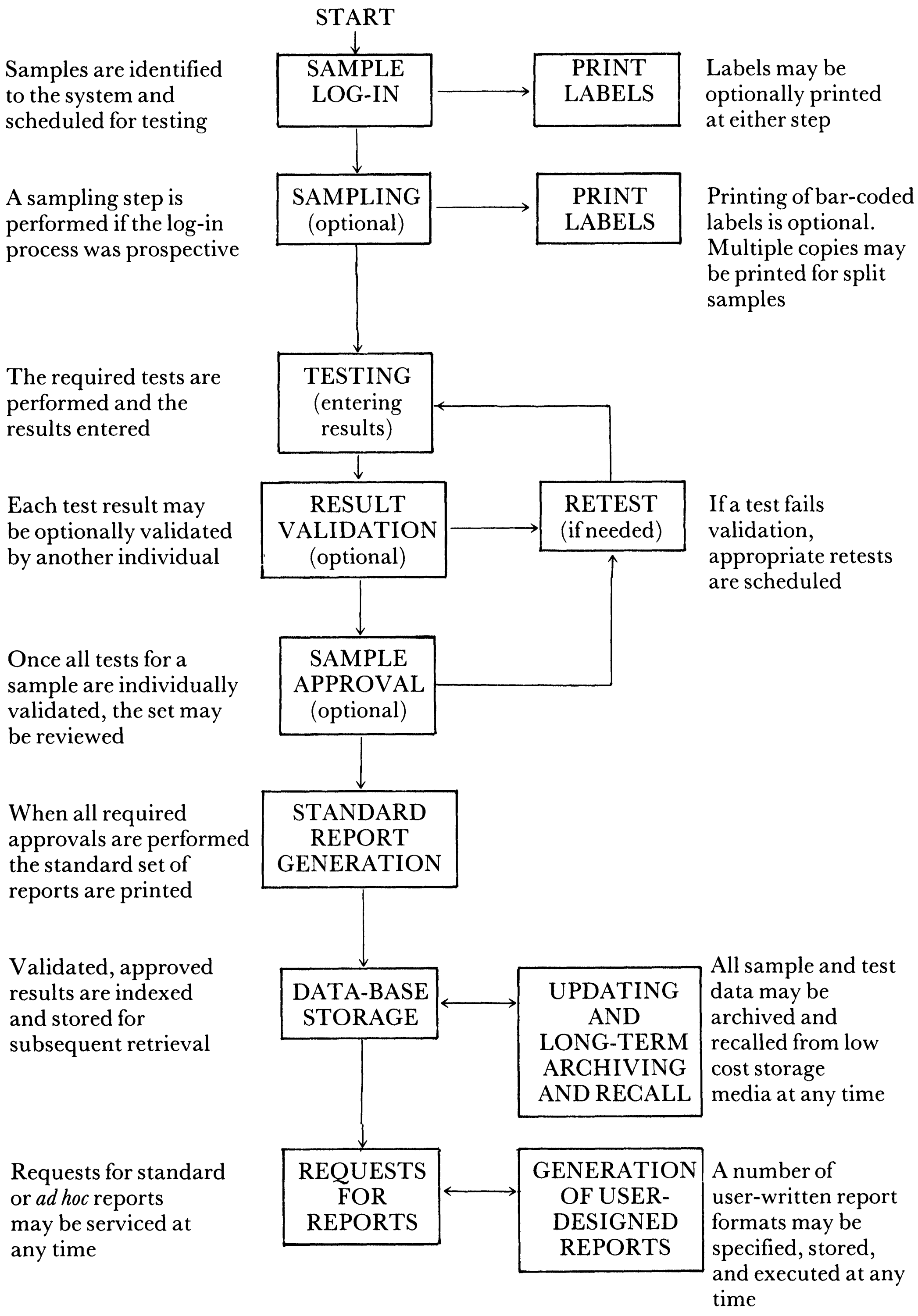

*Figure 1. Operational task sequence.*

When tests require it, Lab Manager will automatically compute secondary parameters from raw values. For example if a test determines density in order to compute other properties such as mass, the user only needs to enter the raw test result and Lab Manager will perform calculations to obtain values of appropriate properties. Calculation procedures are very similar to common algebra and can be easily specified by laboratory personnel without any programming knowledge. Calculations can involve addition, subtraction, multiplication, division and exponentiation.

Lab Manager allows setting up a sample and sequentially performing all tests associated with that sample. However, laboratory personnel will often find it easier to set up and perform one specific analysis for many samples before proceeding to the next test. Lab Manager handles this situation through its RUNSHEET processing feature. This feature groups together all samples scheduled for the same test and displays them on the terminal screen for the user to select samples for testing.

For simplicity, types of test supported by Lab Manager are classified by the characteristics of their results:

(1) Single answer tests: Lab Manager simply presents a test specific question and asks the user to enter the answer (for example yes/no, pass/fail etc.).
(2) Free comment tests: Lab Manager asks for comments or narrative descriptions about the sample. This test functions like a laboratory notebook which records freely worded observations.
(3) Menu tests: Lab Manager will present a menu of choices from which the user selects appropriate answers.
(4) Manual assay: Lab Manager prompts for the quantitative values determined by an assay test. The system allows two sets of limits which define acceptable assay results, as well as specific target values. It records whether the test results fall within laboratory standards. These tests may be set up for an assay of a number of components potentially present in the sample, and for a number of repeated tests (known as replicates). All scheduled replicates must be performed before a test is considered complete.
Automated, analysis: Lab Manager can receive and process the 'raw' data acquired by instrument automation systems such as the CALS Data Acquisition System.

*Validation of test results*

The validation step represents the common practice of requiring one person to review the work of another. The system can be configured so that re-testing can be performed if needed. Since Lab Manager maintains a file of user identification codes and permits various levels of access by assigning a security status to each user, it can prevent validation of a test result, if desired, by the person who performed the test. Validation 'validates' each test. Of course, if such a validation step is inappropriate for a particular laboratory or test it can be omitted from the system or bypassed on a test-by-test basis.

*Approval of all tests for a sample*

The 'approval' step is similar to validation except that approval is performed by laboratory management personnel who are responsible for the quality of sample analysis. While validation applies to tests, approval applies to an entire sample. Even though a test may be validated, a sample is only approved when valid test data yield the approved results. This step may also be omitted from a particular system.

*Generating standard reports*

The report generation phase may take various forms in different laboratories. One important standard report is a 'Certificate of Analysis'. This certificate lists samples together with their tests and results. Although the format of certificate headings and footings is configurable for specific user needs, its unique feature in any situation is the fact that only one official copy can be obtained; every other copy is labelled either as 'preliminary' or 'duplicate'. Such a feature is essential for those laboratories which must conform to specific standards, certify their processes, or control report documents.

Status reports are also available from Lab Manager. These reports display the latest status of each sample logged into the system; the status of any particular sample may be 'awaiting testing', 'approved', etc.

Many other standard reports can be produced by the system on a routine basis. The content and format of those reports are configurable and may be established at the time of system installation and may be modified by users through data-base functions (see below).

*Data-base retrievals*

Perhaps one of the most important components of Lab Manager is the data-base management system, which organizes storage and retrieval of data for all samples logged into the system, whether they are active (still undergoing tests or awaiting validation or approval) or complete (all operations finished). The user may develop retrieval requests for most of the data or specifications in the Lab Manager system. These requests define values which specific fields in the records of samples must contain to be selected for retrieval. The selection criteria may be very diverse and specific, making use of standard logical and relational functions (for example and, not, or, equal to, less than, greater than etc.).

*Data-base report generation*

Any data retrieved from the data-base may be formatted and printed as a report. Lab Manager offers considerable freedom for report design by offering a powerful report generation facility. This facility may be used by anyone without knowledge of programming because instructions are entered with English-like commands. Not only can the user set margins, spacings, headings and footings, but a report may be designed which is printed, labelled, sorted, totalled, and averaged in several ways. Retrieved data may also be plotted in a variety of formats, including histograms and trends using an optional x–y plotter. Multi-colour plotting is directly supported. Plots may be fully labelled and automatically scaled (see figure 2). Additionally, data retrieved by Lab Manager may be stored on a disk file for subsequent processing by user-written programs.

The data-base itself stores frequently used retrievals and report formats by name; Lab Manager will perform the retrieval and print, plot or display the report simply by entering the procedure name. These procedures may be edited and revised at any time by an authorized user.

*Updating and archiving*

Data-base updating and archiving functions are standard features of Lab Manager software. Archiving is usually scheduled weeekly, while updating is normally a daily routine. Lab Manager updates its data continually. Therefore, all data is retrievable virtually as soon as it is entered. Lab Manager's overnight update program

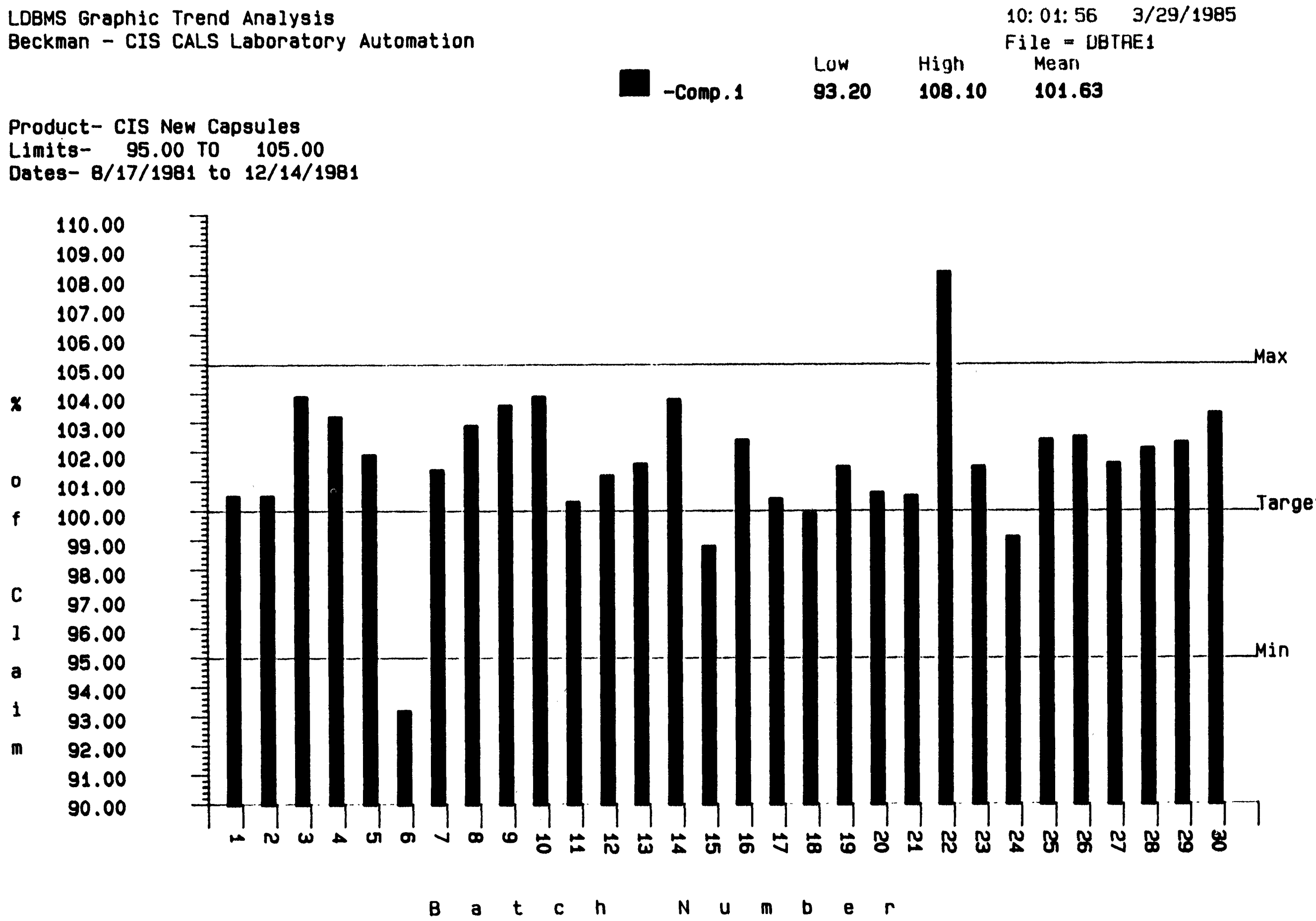

*Figure 2.*

changes the status of samples from 'active' to 'completed', that is approved and reported. Archiving establishes long-term storage of data from completed samples, usually on low cost media such as magnetic tape. Archived data may be recalled for further analysis or reporting at any time. Such recalled data is appropriately marked in the data-base to prevent unauthorized or inappropriate modification.

### Dictionaries

There are several special files which contain the controlling information for use by Lab Manager. Together they define the parameters and procedures which the system uses to perform information processing unique to each laboratory. These files are known as Lab Manager dictionaries and are modified and updated interactively by the laboratory as requirements evolve. The dictionaries are listed and described below:

(1) Test dictionary: defines all test procedures, arranged according to the various test types described above. Stores user prompts, test protocols, units of measurement, and linkages to any required calculation routines or automated analysis files.

(2) Calculation dictionary: defines the procedures for various calculations, including conversational input statements and BASIC-like algebraic computations. A variety of intrinsic functions is provided to simplify calculation definitions. All procedures may be referenced by any or all tests for simplicity, economy and consistency.

(3) Specification dictionary: defines specifications for each product tested in the laboratory. Lists applicable tests, defines result limits and other procedural items for each sample of the product which is to be processed. This dictionary represents the highest level of substance identification. The most important function of this dictionary is to allow automatic assignment and scheduling of routine tests without the user having to individually assign tests to samples of routinely tested products. This feature contributes significantly to laboratory productivity whilst minimizing transcription and errors of omission.

(4) Data-base dictionary: defines the specific procedure for retrieval of data from the data-base, as well as user-written report procedures which instruct the system to format reports. This dictionary may also contain procedures which retrieve and report data or specifications stored in other Lab Manager dictionaries.

(5) Identification dictionary: defines log-on information about each user, including name, password, menu set, and security class. Usually the laboratory system manager maintains this dictionary as employees and authority classes change.

These dictionaries contain all information relevant to the general operation of the Lab Manager system. The information is organized as individual records within each dictionary which define specific operations or functions of Lab Manager. Each record contains defined fields which hold the information. For example, the first field of the identification dictionary contains the name of the user. One of the reasons Lab Manager is so adaptable to various laboratory environments is that many of these fields can be defined to represent the type of data and specifications normally encountered by a particular user.

Therefore, even the prompting messages and headings displayed on the terminal may vary from one laboratory to another, each requesting or presenting data in appropriate terminology.

Laboratories may define additional dictionaries for use in retrievals and reporting as requirements dictate.

### Data management functions

There are many operations which Lab Manager performs upon simple command. These are known as Lab Manager functions and their variety reflects the power and wide usefulness of the system.

### *Sample tracking*

Lab Manager provides extensive facilities to track and control samples throughout the laboratory. Indeed, management of samples and subsequent test results is the primary function and value of the Lab Manager System. Once a sample has been logged-in, its overall status and the status of any associated test may be reviewed at any time. Each major function automatically modifies status, time and date indicators to ensure that reviews are always current. Reports of all samples with any combination of status and date may be displayed or printed on request by any authorized user. These capabilities ensure that high priority, old or problem samples can be quickly isolated and the trouble corrected.

### *Work-list generation*

As a result of the complete sample tracking facilities, production of current work-lists is simple. Most laboratories schedule the work-list reports for after-hours execution in order to have a 'backlog' report at the start of the next working day. Since data for work-list reports is selected with Lab Manager's report generating facility, it may have the format best suited to the laboratory's requirements. In fact, multiple work-list reports may be generated, dividing the work by sample type, test type or any other parameters associated with the sample or tests.

### *Runsheet generation*

When several tests are routinely conducted on samples, it may be convenient to organize result entry by test rather than by sample. The optional RUNSHEET feature allows a work-list to be built by a data-base retrieval of all samples currently pending the test of interest. The work-list may then be modified by addition or deletion or rearranged. If a 'priority' field has been included with either the samples or tests, the work-list may be automatically sorted by ascending or descending priority value.

Once the desired list is generated and optionally modified, the test for any displayed sample may be executed. If the test at the top of the list is selected, only a single keystroke is required to initiate the test.

### *Method management*

In many laboratories, methods and procedures form a major information based investment. The protection and proper treatment of that investment is a real concern. Lab Manager provides many different facilities to manage the information representing methods and procedures with minimal effort and paperwork.

Lab Manager organizes methods, tests and calculations into consistent, accessible formats. Both may be updated and revised with user-friendly conversational programs by an authorized user. Both methods and calculations may be referenced by any sample or test, respectively, to eliminate redundant entry and storage and to promote consistency of use.

Methods and calculations are protected both by Lab Manager's general mechanism of security classes and a password. Any and all revisions are date- and time-stamped automatically and the initials of the user are recorded.

### *Cost accounting facility*

Cost accounting information can be easily derived from Lab Manager, minimizing administrative paperwork. If so configured, an accounting charge number can be associated with each sample at log-in. Similarly, a fixed cost and an elapsed time cost rate may be specified with the definition of each test. Lab Manager automatically records the actual time required to conduct each test. These time and costing values may be combined under the usual formulas to compute total testing costs.

These costs can be sorted and totalled by charge number using the report generator. Detailed invoices reflecting the cost of conducting tests for internal or external clients can be easily generated. Since these invoices are reports produced with the Lab Manager and *ad hoc* report generator, a variety of formats is possible. If necessary, these invoices can be transmitted directly to other computers without ever producing a paper copy.

### Audit trail facilities

Lab Manager automatically records the execution of each function in a simple sequential file. Each record of this file corresponds to a single execution event and is automatically identified with the time, date, user ID and function code. Additional parameters are also included which are specific to each record type. The event log file has numerous uses, ranging from a security tracking mechanism to developing function usage frequency measurements.

Additionally, each test result is automatically stamped with the time, date and user ID as it is entered. Thus, all results may be listed by chronological order of execution should such a review become necessary.

### GLP/GMP compliance

An increasing number of laboratories are interested in complying with good laboratory practices, either due to regulatory oversight or local standards. Lab Manager is specifically designed to operate in and adapt to these types of strict but evolving environments. Consequently, Lab Manager contains many features which automate or assist the laboratory in establishing and maintaining such procedures. Many of the features provided by Lab Manager have already been mentioned and are summarized here.

(1) Security – up to 16 classes of authorization may be defined and redefined by the laboratory.
(2) Audit trail – entry of test results and revision of methods and dictionaries are automatically stamped with the current time, date and identification of the system user. Each system action is recorded on a summarized event log which is also fully identified. All results, including retests, invalid and unapproved results are maintained on the data-base for review. Methods and test procedures are automatically stored with the acquired data or results for reanalysis and audit purposes, independent of routine method or test procedure revision.
(3) Document control – the certificate of analysis and similar reports are automatically titled to indicate 'original' or 'duplicate' status. All reports, including *ad hoc* tabulations, include the time and date of generation.
(4) Procedural conformance – Lab Manager enforces strict compliance with the test-validate-approval sequence, where all tests must be validated and all tests must be both conducted and validated prior to approval. Lab Manager may be configured to require a free text comment when retesting is requested.
(5) Data archiving and recall – all data may be easily archived and subsequently recalled for analysis or reporting. Recalled data is marked automatically to prevent erroneous analysis.
(6) Data indexing and retrieval – all data organized with Lab Manager are indexed and may be rapidly retrieved by up to 79 user-specified fields. Product code, sample ID, test date and user ID are typical, familiar examples of index fields. Specification of a field as a data-base key greatly increases the speed of access. However, data may be retrieved based upon the values of any field with a sequential search. The speed and ease of data retrieval make compliance with audit and review requirements a simple matter.
(7) Time, date and user ID – all test results are automatically stamped with current time, date and the user's unique identification.
(8) Data validation – quantitative test results may be validated against preset limits appropriate to the test and product. If desired, Lab Manager may be configured to require a manual verification step performed by other than the original testing user. Similarly, an approval step spanning all results for all tests for product sample may be required. Generation of exception reports and flagging values which are out-of-limits are a simple matter.
(9) Documentation – the Lab Manager dictionaries provide consistent, uniform, typed documentation for tests, reports, procedures, and security classes. These may be printed for distribution at any time.
(10) Source code – upon execution of the appropriate non-disclosure agreements, Beckman provides users with source code for Lab Manager modules. Although not written into regulations, at least one regulatory agency considers source code availability a significant issue.
(11) Management oversight – use of Lab Manager's centralized data-base, which is updated on-line, and powerful enquiry facilities make status reporting a simple and effective matter with virtually no loss of productivity even when frequently performed.

These features, in concert with reduced manual transcription and computation, significantly increase the accuracy and completeness of the total laboratory information handling process.

### Summary

In summary, Lab Manager provides a full range of laboratory information management services. The system is flexible enough to be useful in all laboratory environments and simple enough for laboratory personnel to operate without any training in computer programming.

